# The role of CRP and Pentraxin 3 in the prediction of systemic inflammatory response syndrome and death in acute pancreatitis

**DOI:** 10.1038/s41598-019-54910-8

**Published:** 2019-12-04

**Authors:** Sebastian M. Staubli, Juliane Schäfer, Rachel Rosenthal, Jasmin Zeindler, Daniel Oertli, Christian A. Nebiker

**Affiliations:** 1 Department of Surgery, University Hospital Basel, Spitalstrasse 21, 4031 Basel, Switzerland; 2 Basel Institute for Clinical Epidemiology and Biostatistics, Department of Clinical Research, University Hospital Basel, Spitalstrasse 12, 4031 Basel, Switzerland; 3 University of Basel, Faculty of Medicine, Klingelbergstr. 61, 4056 Basel, Switzerland; 4 Department of Surgery, Hospital of Aarau, Tellstrasse 25, 5001 Aarau, Switzerland

**Keywords:** Predictive markers, Acute pancreatitis

## Abstract

Pentraxin 3 (PTX3) is an acute phase protein. Our goal was to assess PTX3 as a predictor of systemic inflammatory response syndrome (SIRS), death and disease severity in acute pancreatitis (AP) in comparison to C-reactive protein (CRP) and the APACHE II score. From April 2011 to January 2015, 142 patients with AP were included in this single center post hoc analysis of prospectively collected data at the University Hospital Basel, Switzerland. Disease severity was rated by the revised Atlanta criteria (rAC). Inflammatory response was measured by the SIRS criteria. PTX3, CRP and APACHE II score were measured. Patients median PTX3 plasma concentrations in AP were higher in moderate (3.311 ng/ml) and severe (3.091 ng/ml) than in mild disease (2.461 ng/ml). Overall, 59 occurrences of SIRS or death were observed. In the prediction of SIRS or death, PTX3 was inferior to CRP and APACHE II, with modest predictive discriminatory ability of all three markers and AUC of 0.54, 0.69 and 0.69, respectively. Upon combination of CRP with PTX3, AUC was 0.7. PTX3 seems to be inferior to CRP and APACHE II in the prediction of SIRS or death in AP and does not seem to improve the predictive value of CRP upon combination of both parameters.

## Introduction

Pentraxin 3 (PTX3) is an acute phase protein and a member of the pentraxin family, together with C-reactive protein (CRP, PTX1) and serum amyloid P (PTX2). Although these biomarkers belong to the same family, there are marked structural and functional differences. In contrast to the short pentraxins CRP and serum amyloid P, PTX3 is a long pentraxin^1^. PTX3 is highly conserved throughout evolution and a functional ancestor of antibodies that recognizes and opsonizes pathogens through associated molecular patterns to activate and direct humoral and cellular immune response^2^. Its role is not purely pro-inflammatory, but also anti-inflammatory, which led to the term “yin and yang-role” of PTX3^3^.

PTX3 is directly released by neutrophil granulocytes upon inflammatory stimulus but can also be synthesized de novo by other cells^4^. In contrast, CRP is produced in the liver upon stimulation of Interleukin-6. PTX3 peaks at a maximum level of 200 to 800 ng/ml within 6 to 8 hours, whereas CRP reaches its peak concentration within 24–48 hours of the inflammatory stimulus^5^.

High serum-levels of PTX3 have been linked to the development of systemic inflammatory response syndrome (SIRS) and sepsis, and ultimately fatal outcomes in critically ill patients^6^. Other studies have shown higher PTX3 serum-levels in cardiovascular diseases, malignancies and infections^7^.

In contrast to CRP, PTX3 is not routinely used in daily practice, because it has not been validated for clinical use and routine laboratory testing is not readily available.

The aim of this study was to further elucidate the role of PTX3 as a diagnostic and prognostic marker in acute pancreatitis (AP), a potentially strong inflammatory disease, and compare it to CRP. The role of PTX3 as a predictor of severity in AP has previously been studied in AP, with conflicting results^8–10^. Furthermore, a formal link between PTX3, SIRS and disease severity of AP has not yet been established. The course of AP is highly variable, ranging from mild acute pancreatitis (MAP), moderately severe (MSAP) to severe acute pancreatitis (SAP) with mortality rates from 3% to 30% or even higher if infected necrosis occurs^11,12^. An ideal biomarker to predict the severity of AP at an early time-point has not yet been established and research to find such a biomarker is still active.

Severity prediction in AP plays an important role and many biomarkers, imaging tools and scores have been developed to anticipate the course of the disease. All of them have drawbacks, mainly insufficient precision and labor-intensiveness^13^. In recent years, further insights in the pathogenesis of AP have been gained and two groups of patients with a high risk of mortality could be identified: patients with organ failure (OF) and those with SIRS. SIRS is defined as systemic inflammatory response to a variety of severe clinical insults. The response is manifested by two or more of the following conditions: (1) temperature >38 °C or <36 °C; (2) heart rate >90 beats per minute; (3) respiratory rate >20 breaths per minute or PaCO2 < 32 mm Hg; and (4) white blood cell count >12,000/cu mm, <4,000/cu mm, or >10% immature (band) forms^14^. As most patients who are progressing towards OF in AP display at least two signs of SIRS in AP, SIRS itself is a predictor of OF and mortality in AP^15^. This instance is also reflected in the revised Atlanta classification of 2012 (rAC), where OF has been attributed more importance and SAP is defined by the persistence of OF longer than 48 hours^16^.

The aim of this study was to assess if PTX3, alone or in combination with CRP, could serve as an early marker of SIRS, disease severity and death in AP.

## Results

### Patients

In this post hoc analysis of prospectively collected data, 142 patients with AP were included, out of which seven patients had two episodes of AP during the study period. We considered these patients only once, with their first episode used for all analyses. The patients’ demographic and baseline clinical characteristics are shown in (Table 1). The median age was 57 years, 43% were female and the predominant cause of pancreatitis was biliary (61%). Eighteen percent had a history of pancreatitis without fulfilling the diagnostic criteria for chronic pancreatitis (patients with chronic pancreatitis were excluded from the study). In 41% of all cases, a computed tomography scan (CT scan) was performed. The median time from study inclusion to CT scan was 0 days (interquartile range [IQR] 0–2), the predominant finding being edematous pancreatitis. Endoscopic retrograde cholangiopancreatography (ERCP) was conducted in 21% of all patients with biliary pancreatitis at a median time of 2 days after study inclusion (IQR 1–4). The mean length of hospitalization was 8 days (IQR 6–12, Range 0–133 days).

**Table 1:** Patient characteristics. Data are median (interquartile range) if not stated otherwise.

Characteristic	All patients (n = 142)
Age, years	57 (44, 72)
Female sex, n (%)	61 (43)
Pregnancy, n (%)	1 (1)
Cause of pancreatitis, n (%)	
Alcoholic	33 (23)
Biliary	86 (61)
Other	23 (16)
History of pancreatitis, n (%)	26 (18)
History of rheumatic disease, n (%)	9 (6)
Computed tomography (CT) scan, n (%)	58 (41)
Time from study inclusion to CT scan, days	0 (0, 2)
Findings CT scan^a^, n (%)	
Normal	7 (5)
Edematous pancreatitis	48 (34)
Pancreatic necrosis	10 (7)
Abscess	0 (0)
Pseudocyst	5 (4)
Bleeding	2 (1)
ERCP^b^ (endoscopic retrograde cholangiopancreatography), n (%)	18 (21)
Time from study inclusion to ERCP^b^, days	2 (1, 4)

^a^Multiple findings per patient possible.

^b^Among patients with biliary pancreatitis.

### Severity of pancreatitis and serum concentrations of biomarkers at admission

A total of nine patients were classified as having SAP, 35 MSAP, and 81 MAP according to the rAC. Seventeen patients could not be classified because of missing covariate information, 10 of whom were discharged before the end of the 4-day observation period. They did not suffer from severe pancreatitis according to their discharge summary. From the 35 patients with MSAP, 18 had transient organ failure and 24 suffered from local complications.

**Figure 1:**
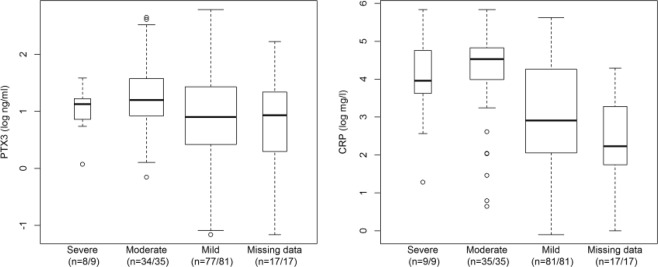
Biomarker levels at study inclusion. The levels of biomarkers at study inclusion among patients with severe, moderate and mild acute pancreatitis according to the Atlanta 2012 criteria as well as among those with missing data. The boxes are drawn with widths proportional to the square root of the number of observations in the four groups. Abbreviatios: PTX3, Pentraxin 3, CRP, C-reactive protein; severe, moderate and mild relate to the severity grades of the revised Atlanta Classification of 2012.

Patients with SAP or MSAP had higher PTX3 and CRP levels than those with MAP or missing covariate information (Fig. 1), while the group of patients with SAP was small and PTX3 only measured in eight out of a total of nine patients.

The ratios of geometric means of PTX3 and CRP levels between patients with MSAP and those with MAP were 1.49 (95% confidence interval [CI] 1.08, 2.07; p = 0.017) and 3.08 (95% CI 1.73, 5.50; p < 0.001). There was no evidence for a difference between MSAP and SAP in CRP as well as PTX3 (Table 2).

**Table 2:** Ratios of geometric means (with 95% confidence intervals) of biomarker levels at the time of inclusion in the study: comparisons between patient subgroups defined according to the Atlanta 2012 classification of acute pancreatitis.

	Main analysis		Sensitivity analysis	
Severe (n = 9) Moderate (n = 35) Mild (n = 81)	Ratio of geometric means (95% CI)	P-value	Ratio of geometric means (95% CI)	P-value
**PTX3**^a^				
Severe vs. mild	1.21 (0.67, 2.18)	0.530	1.22 (0.73, 2.05)	0.450
Severe vs. moderate	0.81 (0.43, 1.51)	0.509	0.97 (0.56, 1.68)	0.916
Moderate vs. mild	1.49 (1.08, 2.07)	0.017	1.26 (0.95, 1.67)	0.109
**CRP**^b^				
Severe vs. mild	2.61 (0.95, 7.14)	0.061	3.66 (1.40, 9.56)	0.008
Severe vs. moderate	0.85 (0.29, 2.47)	0.761	0.70 (0.25, 1.97)	0.497
Moderate vs. mild	3.08 (1.73, 5.50)	<0.001	5.23 (2.99, 9.16)	<0.001

Abbreviations: CI, confidence interval; PTX3, pentraxin 3; CRP, C-reactive protein.

^a^Main analysis – available in 8/9 (89%), 34/35 (97%) and 77/81 (95%) patients with severe, moderate and mild AP; sensitivity analysis – available in 7/9 (78%), 31/35 (89%) and 73/81 (90%) patients with severe, moderate and mild AP after the removal of eight outliers, six with an unusually low PTX3 level (one from the severe AP group, one from the moderate AP group, and four from the mild AP group) and two with an unusually high PTX3 level (both from the moderate AP group).

^b^Sensitivity analysis – available in 8/9 (89%) and 29/35 (83%) patients with severe and moderate AP after the removal of seven outliers with an unusually low CRP level (one from the severe AP group and six from the moderate AP group).

In sensitivity analyses, we excluded a few unusual and potentially influential observations to see whether our analyses were robust with respect to outliers. Overall, the results were comparable to those of the main analyses (Table 2).

### Discriminatory ability of PTX3, CRP and the APACHE II in the prediction of SIRS or death

Fifty-nine (42%) patients developed SIRS or died within four days of study inclusion. Two patients died of sepsis, whose PTX3-levels were 2.5 ng/mL and 2.9 ng/mL. We assumed that the 10 patients who were discharged before the end of the 4-day observation period did not die and showed no SIRS. Therefore, a total of 59 patients showed SIRS with two or more criteria or died and 78 did not. Five (4%) patients had missing covariate information and could not be classified.

**Figure 2:**
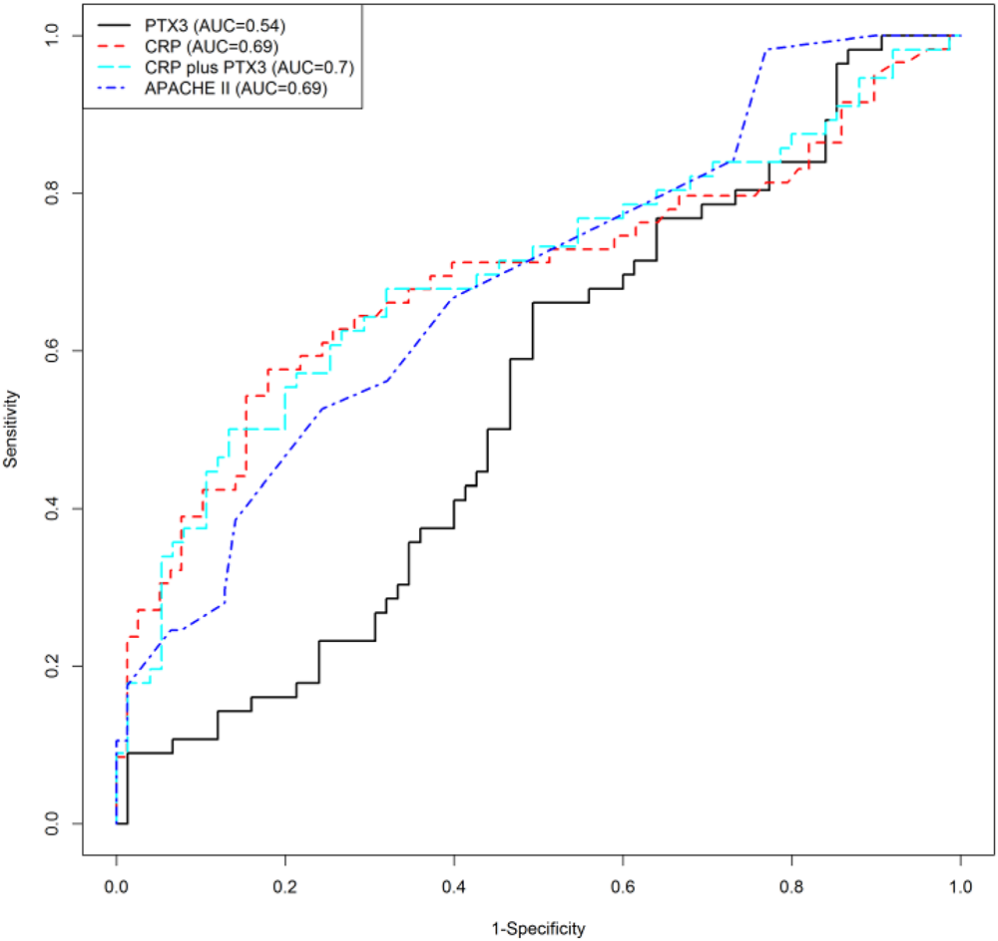
Discriminatory ability of the biomarkers to predict SIRS or death. Abbreviations: PTX3, Pentraxin 3; CRP, C-reactive protein; APACHE II, acute physiology and chronic health evaluation II.

The AUC for the models to predict SIRS or death were weak for PTX3 and modest for CRP and the APACHE II score, with values for PTX3, CRP and the APACHE II of 0.54, 0.69 and 0.69, respectively (Table 3, Fig. 2). Upon combination of CRP and PTX3, AUC was 0.7. The odds ratios per standard deviation increase in log-PTX3 concentration were 1.29 (95% CI 0.91, 1.87; p = 0.168), log-CRP 1.94 (95% CI 1.34, 2.89; p < 0.001) and the APACHE II 2.28 (95% CI 1.5, 3.68; p < 0.001).

**Table 3:** Univariate logistic models for the prediction of SIRS or death within four days of inclusion in the study.

	All patients (n = 137)				Patients without SIRS at study inclusion (n = 97)			
Prognostic variable	N	OR (95% CI)	P	AUC	N	OR (95% CI)	P	AUC
PTX3 (by one SD increase)	131	1.29 (0.91, 1.87)	0.168	0.540	93	1.32 (0.78, 2.37)	0.331	0.525
CRP (by one SD increase)	137	1.94 (1.34, 2.89)	0.001	0.692	97	1.12 (0.67, 1.88)	0.674	0.527
APACHE II (by one SD increase)	135	2.28 (1.50, 3.68)	<0.001	0.685	97	2.09 (1.29, 3.70)	0.006	0.663

Abbreviations: OR, odds ratio; CI, confidence interval; AUC, area under the ROC curve; SD, standard deviation; PTX3, Pentraxin 3; CRP, C-reactive protein; APACHE II, Acute Physiology and Chronic Health Evaluation II.

Biomarker levels were log-transformed before all analyses to normalize their distribution.

At study inclusion, 97 patients showed no signs of SIRS. From this subgroup, 19 developed SIRS or died during the following four days. The discriminatory ability of the biomarkers was comparable, with weaker predictive ability of CRP (Table 3).

## Discussion

Our study suggests that PTX 3 is inferior to CRP and APACHE II in the prediction of SIRS or death in patients with AP. The combination of PTX3 and CRP does not seem to improve the predictive value of those parameters.

The main strength of the study is a relatively high number of included patients in comparison to other similar studies as well as the usage of the latest classification system (rAC) and the prospective data collection of the study. Furthermore, our collective reflects a realistic depiction of the AP patient population as all etiologies of AP were included in our study.

### Limitations

PTX 3 and CRP do not peak simultaneously, but rather sequentially (PTX 3 after 6–8 h, CRP after 24–48 h subsequent to an inflammatory stimulus). In our collective, blood samples were drawn upon hospital admission and the second and fourth day thereafter. Patients with onset of pain less than 96 hours were included. As many patients have difficulty to reliably report the exact time point of onset of pain, we did not record the exact timepoint of onset of symptoms. As a result, the exact timespan between onset of disease and blood sampling is not known. As shown in a substudy of the ALBIOS trial^7,17^, a multicenter, randomized controlled trial to compare the effect of human albumin and crystalloids versus crystalloids alone in patients with severe sepsis, PTX3-values had a tendency to decrease over time, even in cases of severe sepsis. Therefore, it is possible that PTX3 peak levels were missed and therefore underestimated.

Furthermore, ten patients were discharged before completion of the planned four-day observation period. These patients were discharged due to an uneventful recovery and therefore did not suffer from SAP. In few patients, blood samples could not be drawn at the planned time-point or laboratory tests could not be completed for technical reasons. Also, a longer observation period might have been warranted, as some cases of SAP occurs later in the course of disease and have therefore been missed. Another weakness of our study is that the numbers of SAP and MSAP cases were low in comparison to MAP cases. Furthermore, altered PTX3 serum levels have been found in a number of diseases including cardiovascular disease^5^ and chronic kidney disease^18^; however, the exact impact of numerous co-morbidities on serum-levels of PTX3 has not been elucidated in detail yet. It is therefore possible that other illnesses or factors have influenced the serum-levels in our cohort.

As blood samples were drawn at admission, 48 hours and 96 hours after study inclusion, the observation period was too short to study compensatory downregulation of the immune system, known as anti-inflammatory response syndrome (CARS).

### Relation to other studies and Interpretation of the findings

PTX3 has been extensively studied in the recent past, and a link between SIRS and increased PTX3 levels has been described by different authors^6,19,20^. Bastrup-Birk *et al*. demonstrated that patients with SIRS could be differentiated from healthy controls with an AUC of 0.922^19^. In our work, the discriminatory ability of PTX3 at study inclusion for the occurrence of SIRS or death within four days was weak with an AUC of only 0.54.

PTX3-levels in patients with AP have previously been studied, with conflicting results. Deng *et al*.^8^ reported a series of 70 consecutive patients with AP. In their cohort, PTX3 was identified as an independent prognostic marker of SAP. In presence of SIRS, patients with higher PTX3 levels progressed significantly more often to SAP. Also, PTX3 outperformed CRP and APACHE II score as a predictor of SAP with an AUC of 0.897 (PTX3) versus 0.744 (APACHE II score).

Another study by Kusnierz *et al*.^9^ in 2013 revealed similar results. In their study, 40 patients with AP were included. According to the original Atlanta classification, 28 patients had MAP and 12 SAP. Patients with SAP had significantly higher PTX3-levels on day one and five, but not on day three. The authors report that plasma concentrations in patients who subsequently died were elevated on day 5. Possibly, the loss of significance on day three reflects the rapid physiological decline in serum levels of PTX3 after an inflammatory stimulus, and the reappearance of a significant difference on day 5 when local complications arise.

Simsek *et al*.^10^ found in a study including 44 patients (28 MAP, 16 SAP) as well as 30 healthy controls that PTX3 correlated with the diagnosis of AP and CRP, but not with the severity of the disease.

The above-mentioned studies differ from our work. The ratio of SAP to MAP is higher in the previously mentioned studies than in our collective, which might partially explain the different results. Furthermore, the timepoint of blood sampling differs substantially in the mentioned studies: Kusnierz *et al*. measured PTX3 within 12 hours of onset of symptoms. Deng *et al*. within 48 hours of study inclusion, however, patients with onset of pain earlier than 48 hours were included in the study. Simsek *et al*. do not mention the exact timeframe of analysis within the disease onset.

As shown by the substudy of the ALBIOS trial^7^, PTX3-values have a tendency to decrease over time, even in cases with severe sepsis. The authors of this study conclude, that not only the absolute serum levels of pentraxin reflect disease severity, but also the dynamic of the decline of serum levels. In other words, in patients with severe sepsis, PTX3 serum levels decline slower than in those without severe sepsis.

In the literature, average PTX3 serum-values in healthy individuals have been published to be 2 ng/mL [1.95, 2.04] (geometric mean, confidence interval)^21^. Our collective with complete outcome data had slightly higher PTX3-levels with an average of 2.7 ng/mL. Kusnierz *et al*. published serum levels of PTX3 4.0 ng/mL in MAP and 17.2 ng/mL in SAP. Simsek *et al*. published serum levels of PTX3 of 1.846 ng/mL in patients with AP and 0.341 ng/mL in healthy controls. The serum levels in the study published by Deng *et al*. differed substantially with roughly 10’000-fold lower serum parameter levels than in other studies^8^.

### Implications for routine practice and further research

In our study, we were not able to find a clear correlation between severity of disease, inflammatory reaction as measured by SIRS and increased PTX3 levels. When compared to other published studies, the performance of PTX3 to predict SIRS and disease severity was weak in our collective. This raises a fundamental question, whether the value of PTX3 as a marker and predictor of SIRS and disease severity is underestimated by our study or overestimated by other studies. A possible explanation for the differing results in the above-mentioned studies is the fact that PTX3 measurements might show a relevant increase during a considerably shorter timeframe only and hence peak levels are more easily missed than in CRP.

In order to further clarify the role of PTX3 in AP, a larger study is warranted with the emphasis on measuring PTX3 as early as possible after onset of symptoms. Furthermore, a longer observation period would be of high interest, as PTX3 levels could not only be correlated to SIRS, but also the occurrence of CARS, which occurs late in the course of the disease. A larger panel of inflammatory markers and cytokines should also be analyzed alongside PTX3, in order to better understand its role in the immune system and appearing of SIRS and CARS.

For the moment, we cannot recommend introducing PTX3 in daily practice as a marker of disease severity in AP.

## Materials and Methods

### Study design and population

This study was designed as a post hoc analysis of a prospective, single-center, observational cohort-study at the University Hospital Basel, Switzerland. The same patient cohort was analyzed in a previous study by our group^22^. In this previous study, the association of copeptin, pro-atrial natriurietic peptide, proadrenomedullin and cortisol with disease severity in patients with acute pancreatitis and ability to predict organ failure or death was analyzed. Cortisol emerged as the best predictor of organ failure and death in patients with AP.

Patients were recruited during April 2011 to January 2015. Inclusion criteria were newly diagnosed AP as defined by the original Atlanta classification of 1992^23^. Patients were recruited either upon presentation at the emergency department or when the diagnosis of AP was established on the ward.

Further inclusion criteria were written informed consent, age >18 years and onset of abdominal pain before study-inclusion being less than 96 hours. Pregnancy was not considered an exclusion-criterion. Patients were treated according to the emergency standards provided in our hospital (www.emergencystandards.com).

The study protocol of this study was approved by the local ethics committee (Ethikkommission beider Basel, EKBB 281/10, 10.01.2011) and registered on ClinicalTrials.gov (NCT01293318). The data of the included patients were anonymized. All methods were performed in accordance with the relevant guidelines and regulations.

### Clinical assessment

A questionnaire assessing baseline characteristics was filled in by the study personnel and vital parameters were recorded. Only patients with an onset of abdominal pain of less than 96 hours were included, however, the precise timepoint of onset of pain was not recorded. A series of routine blood samples were drawn at the time point of inclusion as well as 2 and 4 days after inclusion. Two additional 7.5 ml tubes (1x serum and 1x EDTA) were taken at study inclusion and day 2. The required parameters to determine the rAC^16^, Ranson-^24^, Acute Physiology and Chronic Health Evaluation II- (APACHE II)^25^ and the modified Marshall scores^26^ as well as the SIRS-criteria^14^ were obtained from electronic health records. Patients were observed during the first four days after study inclusion. In-hospital mortality was also registered. Patients discharged before completing the 4-day observation period remained included in the study with partially missing follow-up data. If blood oxygen saturation was higher than 90%, arterial blood gas analysis was omitted. In those cases, a partial pressure for oxygen of 90 mmHg was assumed and used for further calculation of scores.

The severity of AP was defined according to the rAC^16^. Local complications were defined as: acute peripancreatic fluid collection, pancreatic and peripancreatic necrosis (sterile or infected), pseudocyst and walled-off necrosis in accordance to the rAC.

OF was defined according to the modified Marshall score^26^.

### Assays

Blood samples were taken as described above and immediately centrifuged and stored at −80° Celsius. All measurements were conducted by a professional lab technician (FC) of the University Hospital Basel.

PTX3 serum-levels were measured by using a commercially available Human PTX-3 ELISA Kit (SEK411Hu, Cloud-Clone Corp., TX, USA). The detection limit is 0.113 ng/mL with a detection range of 0.312 to 20 ng/mL (manufacturer’s information). Average serum-levels of PTX3 in healthy individuals were reported in the literature as 2.28 ± 1.33 ng/mL^21^.

CRP serum-levels were measured by turbidimetry.

### Sample size

A formal sample size calculation was conducted on the basis of the primary research question of our previous study^22^. The present study has to be regarded as exploratory in nature and is based on the available data for the cohort of patients with AP.

### Statistical analysis

#### Association of the biomarkers with disease severity

We used linear regression models to assess the associations of PTX3 and CRP at study inclusion with disease severity according to the rAC criteria. Biomarker levels were analyzed by logarithmic transformation. The results are presented as estimated ratios of geometric means (with their 95% confidence intervals).

#### Development and assessment of prognostic models

To assess the prognostic accuracy of PTX3, CRP and the APACHE II score as measured on admittance (day 0) in predicting the combined endpoint of SIRS or death within the first four days after study inclusion, we used logistic regression models and calculated the area under the receiver operating curve (AUC) separately for each prognostic variable. In addition, the results are presented as estimated odds ratios (with their 95% confidence intervals) per standard deviation increase in the log-transformed prognostic variables. These analyses were repeated for the subset of patients without SIRS when they were included in the study. The analyses were also repeated to see if both markers combined (CRP and PTX3) result in an increased prognostic accuracy compared to the APACHE II score or the two markers alone.

#### Statistical software

We used R 3.2.1 (R Foundation for Statistical Computing, Vienna, Austria) for our analyses and graphics and the ROCR and PredictABEL add-on packages for ROC analysis.
